# Overcoming EMT-associated resistance to anti-cancer drugs via Src/FAK pathway inhibition

**DOI:** 10.18632/oncotarget.2397

**Published:** 2014-08-27

**Authors:** Catherine Wilson, Katrina Nicholes, Daisy Bustos, Eva Lin, Qinghua Song, Jean-Philippe Stephan, Donald S. Kirkpatrick, Jeff Settleman

**Affiliations:** ^1^ Department of Discovery Oncology, Genentech, 1 DNA Way, South San Francisco, CA; ^2^ Department of Discovery Protein Chemistry, Genentech, 1 DNA Way, South San Francisco, CA; ^3^ Department of Discovery Nonclinical Biostatistics, Genentech, 1 DNA Way, South San Francisco, CA

**Keywords:** EMT, Src, drug resistance, dasatinib, cancer

## Abstract

Epithelial to mesenchymal transition (EMT) is a key process in embryonic development and has been associated with cancer metastasis and drug resistance. For example, in *EGFR* mutated non-small cell lung cancers (NSCLC), EMT has been associated with acquired resistance to the EGFR inhibitor erlotinib. Moreover, “EGFR-addicted” cancer cell lines induced to undergo EMT become erlotinib-resistant *in vitro*. To identify potential therapeutic vulnerabilities specifically within these mesenchymal, erlotinib-resistant cells, we performed a small molecule screen of ~200 established anti-cancer agents using the *EGFR* mutant NSCLC HCC827 cell line and a corresponding mesenchymal derivative line. The mesenchymal cells were more resistant to most tested agents; however, a small number of agents showed selective growth inhibitory activity against the mesenchymal cells, with the most potent being the Abl/Src inhibitor, dasatinib. Analysis of the tyrosine phospho-proteome revealed several Src/FAK pathway kinases that were differentially phosphorylated in the mesenchymal cells, and RNAi depletion of the core Src/FAK pathway components in these mesenchymal cells caused apoptosis. These findings reveal a novel role for Src/FAK pathway kinases in drug resistance and identify dasatinib as a potential therapeutic for treatment of erlotinib resistance associated with EMT.

## INTRODUCTION

Modeling of acquired drug resistance *in vitro* using tumor-derived cell lines has provided critical insights into the numerous mechanisms underlying the drug resistance that is typically observed in cancer patients undergoing treatment with various kinase-targeted agents. Such studies have revealed several specific genetic mechanisms of acquired drug resistance that have been observed clinically [1, 2]. More recently, non-mutational mechanisms of drug resistance have also been identified. For example, pre-existing EGFR (Epidermal Growth Factor Receptor) inhibitor-resistant cell populations have been observed *in vitro* within a population of EGFR mutant NSCLC cells, indicating heterogeneity within cancer cell populations, including a transiently maintained “drug tolerant persister” (DTP) subpopulation [2]. Other studies have demonstrated small populations of “cancer stem cells” which appear to be intrinsically resistant to anti-cancer agents*,* possibly reflecting elevated drug efflux potential, as has been associated with normal stem cells [3, 4]. In addition, in several studies of kinase-addicted TKI-sensitive cells, “switching” to an alternative kinase dependency has been observed, highlighting the extensive cross-talk among pathways that drive cancer cell survival and the potential for signal redundancy [5, 6].

EMT, a non-genetically determined process observed within tumor cell populations, has also been associated with resistance to various cancer therapeutics, including TKIs [7-9]. In an EGFR mutant NSCLC patient's tumor biopsy, a subpopulation of mesenchymal tumor cells was identified, which subsequently appeared to give rise to resistance to EGFR inhibitor therapy [1]. To model EMT *in vitro*, numerous experimental strategies have been utilized [10-12], and TGF-β-induced EMT has been demonstrated to promote resistance to TKIs [13]. Maintenance of the mesenchymal state following EMT can be regulated by paracrine and autocrine signals [12], and growth factors derived from tumor cells themselves or the tumor stroma have been shown to promote resistance to TKIs through kinase dependency switching [5, 6]. Activation of receptor tyrosine kinases such as AXL has been observed in the context of EMT-associated drug resistance, however, the functional role of AXL in resistance to TKIs remains controversial [9, 14-16].

In this study we sought to identify potential therapeutic vulnerabilities specifically within the mesenchymal, TKI-resistant cell population. Using a small molecule screening strategy, we observed that the mesenchymal cells are broadly resistant to a variety of anti-cancer agents, including several TKIs and chemotherapeutic drugs. However, a small number of agents showed selective growth inhibitory activity against the mesenchymal cells. Dasatinib, an inhibitor of ABL and SRC tyrosine kinases, was the most potent of the tested agents, and additional studies revealed a role for Src/FAK pathway kinases in EMT-associated drug resistance.

## RESULTS

### RTK-addicted cancer cell lines become TKI-resistant upon EMT

**Figure 1:**
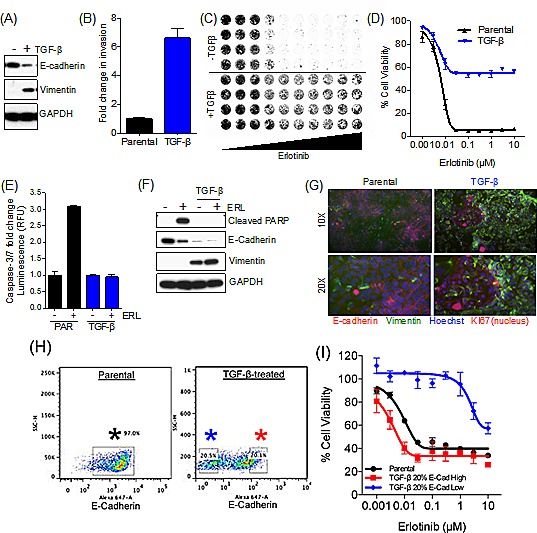
RTK-addicted cancer cell lines acquire TKI resistance upon EMT (A) Immunoblot demonstrating loss of E-Cadherin and an increase in Vimentin expression upon treatment of the lung cancer cell line HCC827 with TGF-β. (B) Bar graph illustrating the enhanced invasion capacity of TGF- β treated HCC827 cells in a 22 hours invasion assay. Error bars represent mean ± SEM. (C) Syto60 assay demonstrating viability of the HCC827 cells following exposure to erlotinib in the parental and TGF-β treated cell line. (D) Cell viability assay demonstrating the effect of erlotinib in HCC827 cells upon EMT. Error bars represent mean ± SEM. IC_50_ values for Erlotinib in HCC827, Parental; IC_50_= 6nM, TGF-β; IC_50_<10μM. (E) Bar graph showing the effect of erlotinib (ERL; 50nM) on Caspase-3/7 activation (24h). (F) Immunoblot showing the effect of erlotinib (ERL; 50nM) on PARP cleavage (apoptosis) after 72h. (G) Immunofluorescence of cell surface E-Cadherin (Red), cyctoplasmic Vimentin (Green), Nuclear Ki67 (Red) and nuclear Hoescht (Blue) in the HCC827 parental and mesenchymal cell lines. (H) FACS analysis demonstrating E-Cadherin expression (Alexa-647) in HCC827 parental and TGF-β-treated cells. Black asterisk: parental cell line E-Cadherin gate; Blue asterisk: TGF-β-treated cells, E-Cadherin 20% low gate; Red asterisk: TGF-β-treated cells, E-Cadherin 20% high gate. (I) Cell viability assay demonstrating the effect of erlotinib in HCC827 parental cells and FACS-sorted TGF-β-treated cells, based on expression of E-Cadherin.

Using continuous TGF-β treatment to promote EMT, we experimentally induced EMT in the HCC827 *EGFR* mutant NSCLC cell line, with previously established sensitivity to the EGFR TKI erlotinib [17]. Exposure of HCC827 cells to recombinant TGF-β for several days resulted in the expected EMT, as assessed by loss of E-Cadherin and gain in vimentin expression (Figure 1A). A mesenchymal phenotype in these treated cells was additionally confirmed by demonstrating their increased invasion capacity (Figure 1B). Next, we compared drug sensitivity of the parental epithelial cells and their mesenchymal derivatives (in the absence of TGF-β). Upon induction of EMT, the HCC827 cells became significantly more resistant to erlotinib (Figure 1 C&D). Erlotinib exposure specifically failed to induce caspase-3/7 activity (Figure 1E) and PARP cleavage (Figure 1F) (markers of apoptosis) in the mesenchymal cells.

Notably, the mesenchymal cells derived following TGF-β exposure were not completely erlotinib-resistant, and 40% of this cell population remained sensitive to drug (Figure 1D). Consistent with that observation, immunofluorescence imaging revealed a subpopulation of epithelial cells (E-Cadherin-positive) within the TGF-β-induced “mesenchymal” population, indicating that not all of the cells had undergone EMT (Figure 1G). Therefore, we sought to determine whether the E-Cadherin-positive subpopulation within the TGF-β-treated population was sensitive to erlotinib by FACS-sorting these cell populations based on E-Cadherin expression (Figure 1H). The FACS-sorted E-Cadherin-positive population was erlotinib-sensitive and exhibited comparable sensitivity to the parental unsorted population, while the E-Cadherin-negative/low population was erlotinib-resistant (Figure 1I). The FACS sorted E-Cadherin-positive population was further exposed to TGF-β, and subsequently underwent EMT, however, this population of cells maintained an E-Cadherin-positive subpopulation of 30-40% (data not shown). Since the TGF-β-treated HCC827 cell population display characteristics of mesenchymal cells, they are hereafter referred to as HCC827 mesenchymal (MES) cells.

**Figure 2:**
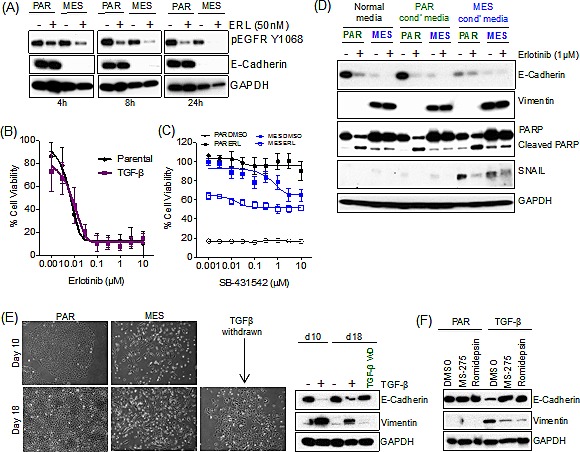
Resistance to erlotinib upon EMT is independent of drug efflux, TGF-β and secreted factors (A) Immunoblot demonstrating the effect of erlotinib (ERL; 50nM) on phospho-EGFR in both the parental (PAR) and mesenchymal (MES) treated cells. (B) Cell viability assay demonstrating the effect of TGF-β in combination with erlotinib for 72 hours in parental HCC827 cells. Error bars represent mean ± SEM. (C) Cell viability assay demonstrating the effect of SB-431542 in combination with erlotinib (1μM) for 72 hours in parental (PAR) and mesenchymal (MES) HCC827 cells. Error bars represent mean ± SEM. (D) Immunoblot demonstrating the effect of erlotinib (ERL; (1μM)) on PARP cleavage (apoptosis) in both the parental (PAR) and mesenchymal (MES) treated cell lines for 72 hours in combination with fresh conditioned (cond’) media following a pre-exposure to conditioned (cond’) media for 3 days. (E) Microscopically observed morphological changes in treated with TGF-β every three days for 21 days and then 10 days removal of TGF-β (left hand side). Immunoblot, demonstrating loss of E-Cadherin and an increase in vimentin expression upon TGF-β treatment and a gain of E-Cadherin and loss in vimentin expression upon withdrawal of TGF-β for 10 days (TGF-β WD) (right hand side). (F) Immunoblot demonstrating no change in E-Cadherin or vimentin expression upon co-treatment of TGF-β with HDAC inhibitors, MS-275 (200nM) or Romidepsin (0.5nM).

We next sought to determine the mechanism of drug resistance following EMT. We first established that erlotinib resistance was independent of drug efflux, as we observed a comparable suppression of phosphorylation of EGFR in the mesenchymal (MES) and the parental (PAR) cells (Figure 2A). TGF-β has both pro-apoptotic and anti-apoptotic properties [18]; therefore, we tested the possibility that recombinant TGF-β was directly promoting erlotinib resistance. Parental HCC827 cells were co-exposed to erlotinib and TGF-β in a 72h viability assay, and there were no detectable differences in the erlotinib IC_50_ (Figure 2B). In addition, cells that were induced to undergo EMT were insensitive to treatment with a TGF-β receptor 1 inhibitor, SB-431542, and co-treatment with erlotinib did not further sensitize the erlotinib-resistant mesenchymal (MES) cells (Figure 2C).

Mesenchymal cells secrete a variety of growth-promoting factors to maintain their mesenchymal state [12], and growth factor-driven resistance has been associated with TKIs, including erlotinib [5, 6]. Therefore, we determined whether conditioned media from HCC827 mesenchymal cells promoted erlotinib resistance upon treatment of the parental cells (Figure 2D). Conditioned media from HCC827 mesenchymal (MES) cells was sufficient to initiate EMT, as indicated by loss of E-Cadherin and gain in expression of Snail in parental HCC827 (PAR) cells; however, vimentin was not detected, indicating that prolonged exposure to conditioned media may be required to promote a complete EMT. However the cells remained sensitive to erlotinib, as indicated by PARP cleavage in a 3-day assay (Figure 2D).

To examine the reversibility of the induced EMT, we sought to determine whether the derived mesenchymal cells would spontaneously revert to a drug-sensitive epithelial phenotype, which would also be indicative of a non-mutational resistance mechanism. We confirmed that these cells were able to revert to an epithelial phenotype upon withdrawal of TGF-β (TGF-β WD) for 10 days (Figure 2E), with restored sensitivity to erlotinib (Figure S1). Considering the reversible nature of the EMT process, we next examined whether the induced EMT was epigenetically regulated. Treatment with the chromatin-modifying agents, MS-275 and Romidespin, both HDAC (histone deacetylase) inhibitors, blocked the EMT process (Figure 2F). These findings demonstrate that the resistance to erlotinib upon EMT is independent of drug efflux, exogenous TGF-β, or secreted growth factors, and is reversibly established through an epigenetic mechanism.

### Mesenchymal HCC827 are sensitive to the Abl/Src kinase inhibitor dasatinib

**Figure 3:**
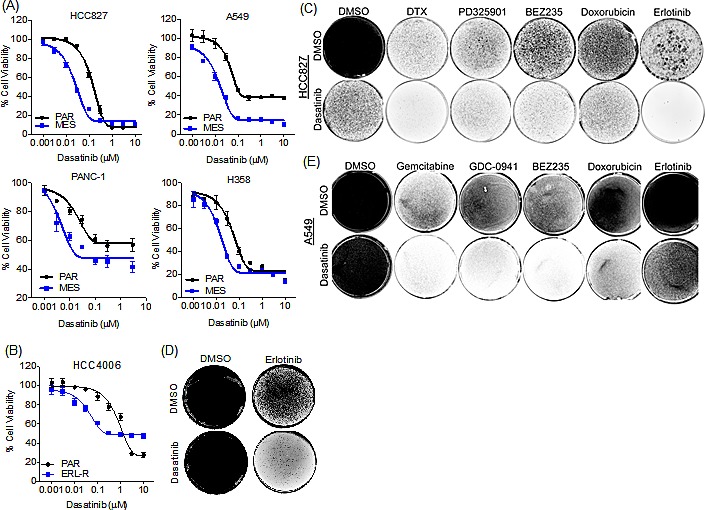
Dasatinib exhibits selective growth inhibitory activity in the mesenchymal cells (A) Cell viability assay demonstrating the effect of dasatinib in parental (PAR) and mesenchymal (MES) lung cancer cell lines, HCC827, A549 and H358 and the pancreatic cancer cell line PANC-1. Error bars represent mean ± SEM. (B) Cell viability assay demonstrating the effect of dasatinib in parental (PAR) and erlotinib-resistant (ERL-R) HCC4006 cells. Error bars represent mean ± SEM. (C) Syto 60 cell staining of HCC827 cells treated with dasatinib (30nM) and erlotinib (60nM), or docetaxel (DTX; 50nM), PD325901 (1μM), BEZ235 (1μM), or doxorubicin (100nM), or in combination every three days for three weeks. (D) Syto 60 cell staining of HCC4006 cells treated with dasatinib (100nM) and erlotinib (1μM), or in combination every three days for ten days. (E) Syto 60 cell staining of A549 cells treated with dasatinib (100nM) and erlotinib (1μM), or gemcitabine (50nM), GDC-0941 (1μM), BEZ235 (1μM), or doxorubicin (100nM), or in combination every three days for three weeks.

To identify anti-cancer agents that are selectively active in the erlotinib-resistant mesenchymal cells, we undertook an unbiased drug sensitivity screen using a panel of 174 established and investigational anti-cancer agents. This analysis revealed that the HCC827 mesenchymal cells were selectively resistant to a variety of other anti-cancer agents, with varying degrees in shift of IC_50_ values relative to the parental cells (Table 1 & S1). However, we did observe that the mesenchymal cells demonstrated increased sensitivity to dasatinib, a dual Abl/Src kinase inhibitor, and not to other distinct ABL/Src inhibitors, such as saracatinib or imatinib. To determine whether this observation could be extended to other TGF-β-induced mesenchymal cell line models, we performed a similar screen with the A549 NSCLC cell line, which also undergoes TGF-β-induced EMT [19]. Upon TGF-β-induced EMT (Figure S2), the A549 cell line becomes resistant to GDC-0941, a PI3K inhibitor, and exhibits resistance to a variety of other anti-cancer agents (Table S2). Consistent with our observation in the mesenchymal HCC827 cell line, the mesenchymal A549 cells demonstrate increased sensitivity to dasatinib (Table S2 and Figure S2). We extended this observation to two additional TGF-β-induced EMT models, the pancreatic cancer cell line, PANC-1 and the NSCLC cell line, H358, as well to an acquired erlotinib resistance model (derived from HCC4006 NSCLC cells) that had undergone EMT independent of TGF-β [20]. In all of these models, dasatinib was more effective at reducing viability in the derived mesenchymal cells relative to their parental counterparts (Figure 3A&B). In addition, a FACS-sorted E-Cadherin-low population of the HCC827 mesenchymal cells demonstrated greater sensitivity to dasatinib than the E-Cadherin-positive population (Figure S3).

**Table 1:** Snapshot of the cell viability of HCC827 parental and mesenchymal cell lines showing IC50 values following 72h exposure to drug Ratio reflects the IC_50_ of mesenchymal/parental cells.

Cell line: HCC827				
Drug	Target	PAR IC50 (μM)	MES IC50 (μM)	Ratio
Erlotinib	EGFR inhibitor	0.006	>10	>1666.667
Gefitinib	EGFR inhibitor	0.003	>2	666.667
Docetaxel	Chemotherapeutic agent	0.032	>10	312.500
PD325901	MEK inhibitor	0.452	>10	22.124
BEZ235	PI3K inhibitor	0.560	>10	17.857
Doxorubicin	Chemotherapeutic agent	0.009	0.039	4.427
Sunitinib	VEGF inhibitor	>10	5.650	0.565
BX912	PDK1 inhibitor	2.160	0.966	0.447
PF-03814735	Aurora kinase inhibitor	3.569	1.235	0.346
BAY 11-7821	NF-κB inhibitor	5.201	1.516	0.291
PHA-739358	Aurora kinase inhibitor	3.382	0.759	0.224
Dasatinib	Abl/Src inhibitor	0.062	0.009	0.145

Since we had detected a low-percentage mesenchymal sub-population that pre-exists within the parental HCC827 cell line (Figure 1G), we next sought to determine whether co-treatment of these cells with a low, ineffective concentration of dasatinib (30nM) and erlotinib could prevent the development of drug-resistance. In HCC827 and HCC4006 cells, co-treatment with dasatinib and erlotinib, prevented the development of resistance to erlotinib (Figure 3C&D), whereas in the A549 cells (EGFR wild-type), which are erlotinib-insensitive, co-treatment with dasatinib and erlotinib did not alter drug sensitivity (Figure 3E). We next sought to determine whether longer-term exposure to other anti-cancer drugs would result in drug resistance, and whether the resistance could be blocked by co-treatment with dasatinib. We selected anti-cancer agents that showed activity selectively in the parental cell line but were ineffective on the mesenchymal cells, based on the cell line screening data (Table 1, S1&S2). In the parental HCC827 cells, drug-resistant cells were generated following exposure to the chemotherapeutic agents docetaxel and doxorubicin or the kinase inhibitors PD325901 (MEK) or BEZ235 (PI-3 kinase) at a relatively high concentration of drug over a period of several weeks (Figure 3C). Co-treatment with dasatinib significantly reduced the number of resistant cells to several tested anti-cancer agents. Similar observations were made in the A549 parental cells, where dasatinib co-treatment decreased the emergence of resistant cells (Figure 3E). In addition, the HCC827 cells remaining following dasatinib treatment alone (Figure 3C) are sensitive to erlotinib, suggesting that the dasatinib-resistant sub-population of cells were epithelial cells (data not shown). These findings suggest that dasatinib co-treatment can prevent the emergence of drug resistance in epithelial cancer cell populations by targeting the innately more drug-resistant mesenchymal cell subpopulation.

### Src/focal adhesion kinase signaling is required for EMT-associated drug resistance

**Figure 4:**
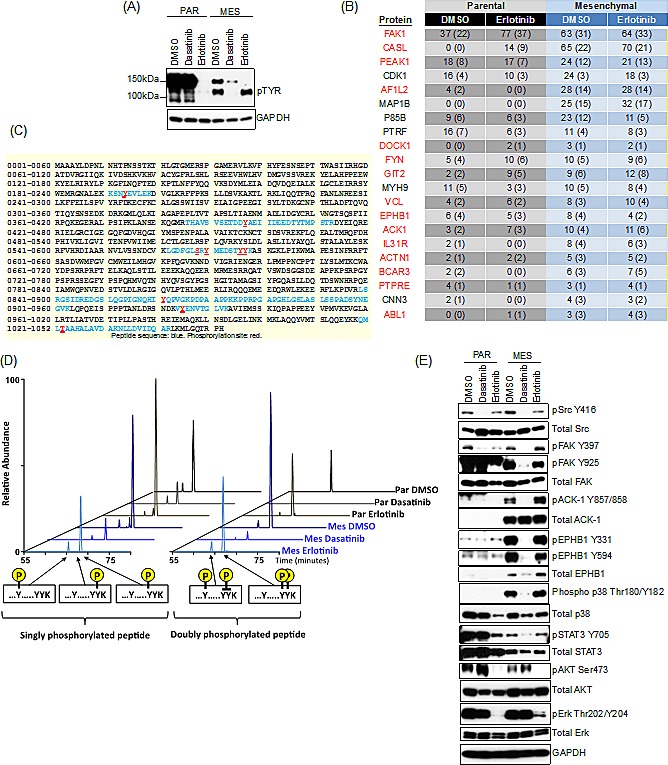
Src/FAK and associated proteins are selectively activated in the mesenchymal cells, and are suppressed by dasatinib (A) Immunoblot demonstrating suppression of a phospho-pY band at 140kDa following treatment with dasatinib (30nM) but not erlotinib (50nM) in the mesenchymal HCC827 cells at 24h. (B) Table of peptide spectral matches (PSMs) total (unique) for phosphotyrosine peptides following erlotinib (50nM) treatment for 24h, demonstrating the most significant changes in phosphorylation in the HCC827 mesenchymal cells. Highlighted in red are those kinases associated with Src/FAK signaling. (C) Mass spectrometry-derived peptide sequence coverage of FAK after phosphotyrosine containing PSMs mapping to FAK. Identified peptide sequences are noted in blue and confidently localized phosphorylation sites are denoted in red. (D) Extracted ion chromatograms of various singly and doubly phosphotyrosinated peptides from sites Y570/Y576/Y577 of FAK showing relative intensities of each species across experimental conditions. (E) Immunoblot demonstrating suppression of phospho-FAK, pACK-1, pEPHB1, phospho-p38 and phospho-STAT3 following treatment with dasatinib (30nM) and not erlotinib (50nM) in the mesenchymal HCC827 cells at 24h.

To establish the mechanistic basis for dasatinib sensitivity in the mesenchymal cell population, we used KinomeView™ western blot analysis to profile the phosphorylation differences between parental and mesenchymal HCC827 cells in the presence and absence of erlotinib to identify the kinase(s) responsible for resistance in the mesenchymal cells. KinomeView™ blots to detect tyrosine phosphorylation revealed major differences following drug exposure in the derived mesechnymal cells compared to the parental cells (Figure 4A). Specifically, we observed a prominent phospho-tyrosine band at approximately 140kDa that was effectively suppressed by erlotinib in the parental cells but not in the mesenchymal cells. Significantly, phosphorylation of a protein at this same molecular weight is suppressed by dasatinib in the mesenchymal cells.

Mass spectrometry analysis, was performed following immunoaffinity enrichment of pTyr-containing peptides and several unique phosphopeptides were differentially observed upon EMT or following drug exposure (Figure 4B and S4). Among these, the non-receptor tyrosine kinase FAK (focal adhesion kinase) was one of the most prominent differentially detected phosphoproteins, with a total of 47 unique pTyr peptides represented in the analysis (Figure S5). In addition, several proteins associated with Src/FAK signaling were also identified, such as EPHB1 (RTK that can activate FAK, Src and CASL) [21], ACK-1 (a non-receptor kinase activated by Src and EGFR) [22], CASL (integrin signaling adaptor) [23], BCAR3 (CAS and Src binding protein) [24], VCL (cytoskeletal protein that regulates focal adhesion) [25] and ABL1 (involved in cell adhesion via phosphorylation of CASL, CRK, CRKL and BCAR1) [26] (Figure 4B and S4).

The differential FAK phosphorylation was prominently at the Y576/Y577 and Y925 sites (Src/FAK recruitment activation) [27] and to a lesser extent at the Y397 autophosphorylation site (Figure 4C). Doubly phosphorylated kinase peptides were generally much less abundant than their singly phosphorylated counterparts in the parental cells; however, they were elevated in the mesenchymal cells, implicating “amplified” FAK signaling in those cells (Figure 4D). Consistent with the mass spectrometry analysis, differential phosphorylation of FAK was observed following erlotinib treatment in both the parental and mesenchymal cells (Figure 4E). In parental cells, but not in the mesenchymal cells, erlotinib suppressed phospho-FAK at the Y397 and Y925 sites (Figure 4E) and not at the Y570/Y576/Y577 sites (Figure 4D). Whereas, dasatinib suppressed phospho-FAK in the mesenchymal cells at each of the phosphotyrosine sites (Figure 4D&E). The mesenchymal cells did not demonstrate sensitivity to FAK inhibitors, PF-562271 or PF-573228, and only demonstrated modest sensitivity to PF-03814735 (Table 1, S1&S2); however, dasatinib treatment caused a more pronounced and prolonged decrease in pFAK than the tested FAK-selective inhibitors (Figure S6). Other kinases identified from the proteomic analysis that are associated with Src/FAK signaling, EPHB1 and ACK-1, were only detected in the mesenchymal cells, and were dephosphorylated by dasatinib and not by erlotinib. Notably, Src kinase itself was not differentially phosphorylated following drug treatment (Figure 4E). Signaling pathways downstream of EGFR, such as PI(3)K and MAPK were comparably suppressed by erlotinib in the parental and mesenchymal cells, which remained unperturbed by dasatinib (Figure 4E). However, downstream Src/FAK survival signals including phospho-STAT3 (Tyr705) and phospho-p38 (Thr180/Tyr182) were decreased by dasatinib but not by erlotinib in the mesenchymal cells. Phosphorylated p38 was elevated in the mesenchymal cells compared to the parental cells; however, the mesenchymal cells were resistant to two p38 MAPK inhibitors, SB202190 and SB220025 (Table S1), suggesting that suppression of the entire Src/FAK pathway is required to decrease cell viability in the drug-resistant mesenchymal cells (Figure 4E).

### EMT-associated drug resistance is dependent on EPHB1/FAK/ACK-1 proteins

**Figure 5:**
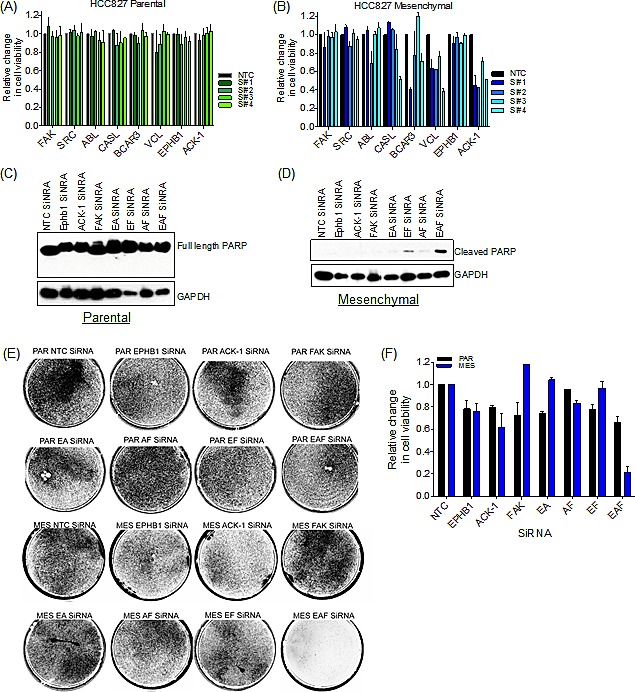
Combination knock-down of FAK/ACK-1/EPHB1 selectively kills resistant mesenchymal cells (A) Bar graph demonstrating cell viability of parental HCC827 cells upon siRNA knock-down of individual kinases or a non-targeted control (NTC). Error bars represent mean ± SEM. (B) Bar graph illustrating cell viability of mesenchymal HCC827 cells following siRNA knockdown of individual kinases or a non-targeted control (NTC). Error bars represent mean ± SEM. (C-F) siRNA knockdown of *FAK*, *ACK-1* and *EPHB1* individually or combined. *EPHB1* and *ACK-1* combined siRNA (EA), *EPHB1* and *FAK* combined siRNA (EF), *ACK-1* and *FAK* combined siRNA (AF) and *EPHB1*, *ACK-1* and *FAK* combined siRNA (EAF). siRNA oligo #4 used in all cases. (C) Immunoblot demonstrating lack of PARP cleavage (apoptosis) in parental HCC827 cells following siRNA knock-down or a non-targeted control (NTC) for 72h. (D) Immunoblot demonstrating PARP cleavage (apoptosis) in mesenchymal HCC827 cells following siRNA knock-down or non-targeting control (NTC) for 72h. (E) Syto 60 cell staining of parental (PAR) and mesenchymal (MES) cells upon siRNA knock-down (double transfection) or a non-targeted control (NTC) for 6 days. (F) Bar graph demonstrating cell viability upon siRNA knockdown (double transfection) or a non-targeted control (NTC) for 6 days. Error bars represent mean ± SEM.

To directly examine a functional requirement for Src/FAK pathway components in mediating drug resistance associated with EMT, we used RNAi to deplete several of the pathway components that were observed to be differentially phosphorylated by mass spectrometry analysis (Figure 4B and S7). Single gene knockdown of FAK, ABL, CASL, BCAR3, Vinculin (VCL) and EPHB1, had little effect on cell viability in either the parental (Figure 5A) or mesenchymal cells (Figure 5B). Similarly, Src depletion did not affect cell viability in either the parental or mesenchymal cells (Figure 5A&B). However, knockdown of ACK-1 decreased cell viability by approximately 50% in the mesenchymal cells (Figure 5B and S7B) with 3 out of the 4 tested siRNAs, with no observed effect in the parental cells (Figure 5A).

Since knockdown of the various individual proteins identified by mass spectrometry analysis did not affect cell viability of the mesenchymal cells, and ACK-1 knockdown was not sufficient to completely kill the mesenchymal cells, we next sought to determine whether simultaneous knockdown of the key kinases that were observed to be differentially phosphorylated in these cell populations could specifically kill the drug-resistant mesenchymal cells. For this analysis, we included FAK, since it was the most abundantly differentially phosphorylated kinase, as well as EPHB1 and ACK-1, since they were only expressed in the mesenchymal cells. In parental cells (PAR), either single or combination knockdown of FAK, ACK-1 or EPHB1 (Figure S8A), failed to induce apoptosis as indicated by PARP cleavage (Figure 5C) or altered cell viability (Figure 5E&F). Conversely, in the mesenchymal drug-resistant cells, simultaneous knockdown of EPHB1, ACK-1 and FAK (EAF) (Figure S8B), resulted cleaved PARP (Figure 5D) and substantial loss of cell viability (Figure 5E&F). These findings suggest that multiple Src/FAK pathway kinases are required to maintain the viability of the mesenchymal state, consistent with the ability of the multi-targeted kinase inhibitor dasatinib to selectively impact viability of these largely drug-resistant cells.

### Dasatinib suppresses mesenchymal cell line tumor growth *in vivo*

**Figure 6:**
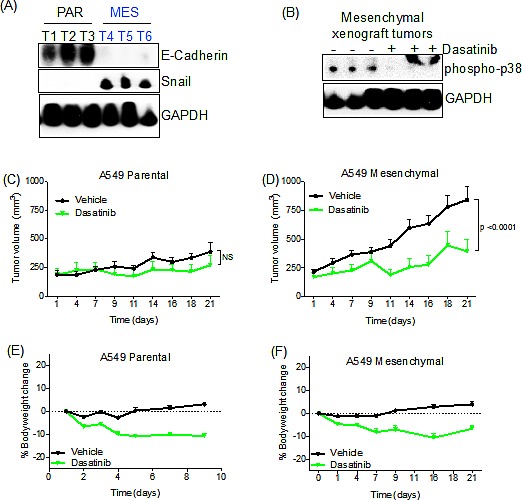
Dasatinib retards the growth of A549 mesenchymal tumor cells *in vivo* (A) Immunoblot demonstrating E-Cadherin and Snail expression in A549 parental (PAR) and mesenchymal (MES) xenografts tumors. (B) Immunoblot demonstrating suppression of phospho-p38 in dasatinib-treated mesenchymal xenografts tumors. A slower-migrating non-specific band is detected in the last two lanes. (C) Tumor growth assay showing the anti-tumor effect of dasatinib in parental A549 xenografts. Mice were treated as described in Experimental Procedures. Difference between dasatinib and vehicle group (NS, p=0.0555) was calculated using two-way ANOVA. Error bars represent the mean ± SEM. (D) Tumor growth assay showing the anti-tumor effect of dasatinib in mesenchymal A549 xenografts. Mice were treated as stated in experimental procedures. Difference between dasatinib and vehicle group (p<0.0001) were calculated using two-way ANOVA. Error bars represent the mean ± SEM. (E) Percentage of body weight change in dasatinib-treated mice bearing A549 parental xenografts. Error bars represent the mean ± SEM. (F) Percentage of body weight change in dasatinib-treated mice bearing mesenchymal A549 xenografts. Error bars represent the mean ± SEM.

To extend the cell line findings to an *in vivo* tumor context, we performed tumor xenograft studies in mice using the parental A549 and derived TGF-β-treated mesenchymal cell lines. The HCC827 mesenchymal cell line failed to grow as a xenograft and therefore could not be used for *in vivo* analysis (data not shown). We first determined whether the TGF-β-induced mesenchymal A549 cells retained their mesenchymal phenotype *in vivo*. Parental A549 and TGF-β-induced mesenchymal cells were each implanted subcutaneously in the flanks of nu/nu mice. Tumors were harvested once they reached 150-200mm^3^, and were found to express significantly lower levels of E-Cadherin and elevated expression of SNAIL compared to the parental xenografts (Figure 6A). We were unable to detect vimentin in these cells (data not shown). Next, to confirm that dasatinib could suppress downstream Src/FAK signaling *in vivo*, we determined that dasatinib treatment effectively suppressed phospho-p38 activity in these tumors (Figure 6B). We then assessed the ability of dasatinib to retard tumor growth in both the parental and mesenchymal cell lines. Tumors were allowed to grow until they reached 150-200mm^3^, after which the mice received dasatinib treatment. Mice bearing A549 parental xenografts grew at a significantly reduced rate relative to the A549 mesenchymal xenografts (Figure 6C,D). Upon dasatinib treatment, A549 mesenchymal xenografts showed significant tumor growth suppression (Figure 6D; p<0.0001). This effect was not observed in the A549 parental xenografts, as tumors in dasatinib-treated animals grew at a similar rate to those in vehicle-treated animals. Notably, the parental xenografts grew at a modestly slower rate compared to the mesenchymal xenografts, potentially contributing to the observed effects of drug treatment on tumor growth inhibition. We observed only a slight decrease in body weight, suggesting that the observed tumor regression is not due to dasatinib toxicity (Figure 6E,F). These observations support the potential *in vivo* utility of dasatinib for a subset of human tumors demonstrating drug resistance associated with a mesenchymal phenotype.

## DISCUSSION

During the genesis of epithelial cancers, relatively rare populations of cells with mesenchymal traits are believed to contribute to tumor initiation, recurrence, metastasis and drug resistance [1, 28-30]. These mesenchymal cells tend to be more resistant to a variety of anti-cancer agents when compared to the bulk tumor compartment of epithelial cells, and therefore present a major challenge to the successful outcome of clinical treatment. Consequently, combination drug treatments that include agents that target the mesenchymal subpopulation of cells may provide a strategy to overcome some types of drug resistance.

Our previous findings revealed a subpopulation of cancer cells that displayed a distinct “cancer stem-like” phenotype with an altered chromatin state, and increased sensitivity to an IGF-1R TKI and HDAC inhibitors [2]. However, the TGF-β-induced mesenchymal cells described here were found to be resistant to IGF-1R TKI and HDAC inhibitors, indicating a distinct mechanism of drug resistance from that seen upon EMT. Our findings demonstrate that the mesenchymal tumor cells are sensitive to dasatinib treatment and that co-treatment of EGFR mutant epithelial lung cancer cells with dasatinib and erlotinib could prevent the emergence of erlotinib resistance. Notably, the combination of erlotinib with dasatinib in advanced lung cancer patients is well tolerated, with some evidence of anti-tumor activity in previously chemotherapy treated patients with advanced NCSLC [31]. However, selection of patients for this co-treatment regimen based on *EGFR* mutation status and expression of mesenchymal markers such as vimentin, may yield greater benefit for this specific patient population.

Targeting Src/FAK pathway kinases in solid tumors has been considered an attractive therapeutic approach by virtue of potential effects on the tumor microenvironment via inhibition of Src family kinase [32]. The Src/FAK pathway kinases are core components of signaling initiated by growth factors, integrins and cytokine receptors to activate downstream signaling cascades such as Ras/Raf/MAPK, PI3K/AKT and STATs [27, 33-35] that control tumor growth [36] and other cellular process such as cell motility, adhesion [27] and EMT [37]. Src/FAK and EGFR converge on common downstream pathways [38], which may account for the observed antitumor activity in patients with advanced NSCLC co-treated with inhibitors of both signaling pathways [31].

Dasatinib is an oral multi-BCR/ABL and Src family tyrosine kinase inhibitor approved for the use in patients with chronic myelogenous leukemia (CML) after imatinib treatment [39, 40] and in patients with Philadelphia chromosome-positive acute lymphoblastic leukemia (Ph+ALL) [41]. More recently it has been shown to be a multi-kinase inhibitor targeting c-Kit, EPH, PDGFR and FAK [42, 43]. Src/FAK signaling has been shown to induce E-Cadherin internalization during cancer progression, in turn promoting EMT and tumor cell motility [44], and EPH's have been shown to be elevated in mesenchymal cells [45]. Therefore targeting Src/FAK and associated kinases with dasatinib in mesenchymal cells may define a useful therapeutic approach.

We observed differential FAK signaling in response to erlotinib in mesenchymal cells compared to epithelial cells. FAK inhibitors themselves did not demonstrate sensitivity in the mesenchymal cells, which may in part be due to their lack of sustained inhibition as compared to dasatinib. In addition, FAK knockdown did not result in cell death of the mesenchymal cells, indicating that FAK alone is not sufficient to perturb the viability of mesenchymal cells. Targeting pathway components downstream of Src/FAK signaling, such as p38, also failed to impact cell viability of the mesenchymal cells. These findings suggest that targeting the key upstream components that are differentially activated upon EMT may be required. We observed that EPHB1 and ACK-1 expression is induced upon EMT, and combination knockdown of EPHB1, ACK-1 and FAK specifically promoted cell death in the mesenchymal cells, to a similar degree as observed following dasatinib treatment. These observations suggest that combined target inhibition of each of the three kinases identified here is required to significantly decrease the viability of mesenchymal cells.

In summary, our findings highlight the utility of TGF-β-induced EMT models of TKI-addicted cancer cells to identify potential therapeutic vulnerabilities associated with this otherwise largely treatment-refractory subpopulation of tumor cells. Such pre-clinical approaches may prove valuable in establishing combination treatment paradigms for tumors that intrinsically display a mixed epithelial-mesenchymal phenotype, or have become more mesenchymal in character during the acquisition of drug resistance.

## METHODS

### Human cancer cell lines

Human cancer cell lines were obtained from American Type Culture Collection or Deutsche Sammlung von Mikroorganismenund Zelkulturen, expanded, and stored at early passage in a central bank. Cells were tested and authenticated by SNP genotyping (see Supplementary Procedures for details). Cell lines were maintained at 37^o^C in a humidified atmosphere at 5% CO_2_ and grown either in RPMI 1640 supplemented with 5% fetal bovine serum (Gibco), 50 U/ml penicillin, and 50 μg/ml streptomycin. To induce EMT, cells were treated with 2ng/ml of rh-TGF-β1 every three days over a two to three week period.

### Inhibitors

Erlotinib and dasatinib were from LC laboratories. Docetaxel and doxorubicin were from Sigma. PD325901 and BEZ235, were from Selleck Chemicals. GDC-0941 was synthesized at Genentech. Gemcitabine was from Toronto Research. TGF-β1 was from R&D Systems Inc. Additional drugs used in the screen are described in Table S3.

### Immunoblotting

Cell lysates were collected using Nonidet-P40 or radioimmunoprecipitation (RIPA) lysis buffer, supplemented with HALT protease and phosphatase inhibitor cocktail (Thermo Scientific), and immunodetection of electrophoresis-resolved proteins was performed using standard protocols. The E-Cadherin, vimentin, Snail, phospho-AKT, total AKT, phospho-p38, total p38, phospho-pERK, total ERK, phospho-STAT3, total STAT3, phospho-Src, total Src, phospho-pFAK, total pFAK, phospho-ACK-1, total pEPHB1, cleaved PARP, pTyr-1000, and GAPDH antibodies were from Cell Signaling Technology. Total ACK-1, phospho-EPHB1 antibodies were from Abcam, and the PARP antibody was from eBioscience. Kinomeview immunoblotting was performed using the pTyr-1000 antibody from Cell Signaling Technology.

### Cell invasion assay

Pre-labeled DiIC12(3) BD Bioscience cell suspensions in serum-free RPMI media were added to the apical chamber of the BD BioCoat™ Tumor Invasion System, 8μm, from BD Biosciences at 3 × 10^5^ cells per chamber. RPMI containing 10% FBS was added as a chemoattractant to the bottom chamber. Chambers were incubated for 20h at 37^o^C, 5% CO_2_, and invasion of fluorescent cells was determined at a wavelength of 549/565 nm (Ex/Em) using the Molecular Devices SpectraMax® M5.

### Cell Viability

Cell viability was assessed using the CyQUANT® Direct Cell Proliferation Assay purchased from Life Technologies. Cells (3000 or 750 per well) were seeded into 96 or 384-well plates, respectively, and allowed to adhere overnight in the absence of TGF-β. They were then exposed to a range of drug concentrations for a 96-well plate. After 72h, CyQUANT® was added per manufacturer's instructions. Cell viability was determined by fluorescence measurement using a 2104 EnVision reader (PerkinElmer). For 6 cm dishes, cells were fixed in 4% formaldehyde, stained with the nuclear fluorescent dye SYTO 60 from Life Technologies, and images were collected by fluorescence measurement using a SpectraMax M5 microplate reader.

### Caspase-3/7 assay

Caspase-3/7 activity was assessed by the Caspase-Glo® 3/7 Assay (Promega). Cells (3000 per well) were seeded, allowed to adhere overnight and exposed to erlotinib (50nM) for 24h in the absence of TGF-β. Caspase-Glo® 3/7 was added to the cells and incubated for 30 minutes at room temperature per manufacturer's instructions. Caspase activity was measured by luminescence measurement using a 2104 EnVision reader (PerkinElmer).

### FACS analysis/sorting

Cells were collected and washed twice in PBS/0.2% serum, and incubated with E-Cadherin from cell signaling, at 1:50, 4^o^C for 1 h. The cells were washed twice in PBS/0.2% serum, resuspended in PBS and sorted on a FACSAria from BD Biosciences.

### Immunoaffinity enrichment and MS analysis

Lysates were collected in buffer containing 20mM HEPES pH 8.0, 9M urea, 1mM sodium orthovanadate, 2.5mM sodium pyrophosphate and 1mM β-glycerophosphate. Immunoaffinity enrichment of pTry phosphopeptides and MS analysis was carried out using PTMscan reagents and protocols (Cell Signaling Technology, Danvers, MA) [46, 47]. Enriched phosphopeptide samples were analyzed on an LTQ-Orbitrap Elite mass spectrometer (ThermoFisher Scientific, San Jose, CA). Samples were injected onto a 0.1 × 100 mm C18 column packed with 1.7 μm BEH-130 material and separated by NanoAcquity UPLC (Waters, Milford, MA) using a standard water/acetonitrile/formic acid gradient. High resolution Orbitrap full MS scans (MS1) were acquired on monoisotopic, charge state defined precursors (*z* > 1) at 60K resolution and data dependent MS/MS spectra acquired in the dual-linear ion trap on the top15 most abundant ions in a data dependent manner. Spectral data were searched using Mascot against a concatenated target-decoy protein sequence database (Uniprot v2011_12) considering oxidized methionine (+15.9949) and phosphorylated serine, threonine or tyrosine (+79.9663) as variable modifications and carbamidomethylated cysteine (+57.0214) as a fixed modification. Peptide spectral matches were serially filtered to a 5% and 2% false discovery rates at the peptide and protein levels, respectively and site localization assessed using AScore [46].

### RNA interference

Transient knockdown of *FAK, ACK-1* and *EPHB1* gene expression was achieved by transfection using ON-TARGETplus siRNA at 12nM (Dharmacon) and Dharmafect 1 (Invitrogen) in the absence of TGF-β. ON-TARGETplus Non-targeting Pool siRNA (Dharmacon) was used as the control.

### Immunofluorescence

Cells were fixed with 4% paraformaldehyde and fluorophore-conjugated antibodies to Vimentin, Ki67 or E-Cadherin from Cell Signaling and were incubated overnight at 4^o^C. Nuclei were stained with Hoechst. Image acquisition was achieved using InCell 2000.

### Xenograft studies

All procedures were approved by and conformed to the guidelines and principles set by the Institutional Animal Care and Use Committee of Genentech and were carried out in an Association for the Assessment and Accreditation of Laboratory Animal Care (AALAC)-accredited facility. Five million A549 parental or mesenchymal cells (suspended in a 1:1 mixture of HBSS/Matrigel) were inoculated in the right flank of Nu/Nu nude mice (Charles River Laboratories). When tumors reached a volume between 150-200 mm^3^, mice were treated with either vehicle control, or dasatinib (10 mg/kg, 5 days/week, via IP injection). Tumors were measured three times weekly using digital calipers (Fred V. Fowler Company) and tumor volumes were calculated using the formula (*L* × (*W* × *W*))/2. Differences between the vehicle and dasatinib groups were determined using two-way ANOVA.

## SUPPLEMENTARY MATERIAL FIGURES AND TABLES


