# Supplementary material for: Overcoming EMT-associated resistance to anti-cancer drugs via Src/FAK pathway inhibition
Source: Oncotarget. 2014 Aug 27;5(17):7328–41. doi: 10.18632/oncotarget.2397 (PMC4202126; doi:10.18632/oncotarget.2397)
Supplement: Supplementary file 1 [file oncotarget-05-7328-s001.pdf]

Overcoming EMT-associated resistance to anti-cancer drugs via Src/FAK pathway inhibition

Supplementary Material

Cell line authentication/quality control:

Short Tandem Repeat (STR) Profiling

STR profiles were determined for each line using the Promega PowerPlex 16 System. This was performed once and compared to external STR profiles of cell lines (when available) to establish cell line ancestry. Loci analyzed: Detection of sixteen loci (fifteen STR loci and Amelogenin for gender identification), including D3S1358, TH01, D21S11, D18S51, Penta E, D5S818, D13S317, D7S820, D16S539, CSF1PO, Penta D, AMEL, vWA, D8S1179 and TPOX.

SNP fingerprinting:

SNP genotypes are performed each time new stocks are expanded for cryopreservation. Cell line identity is verified by high-throughput SNP genotyping using Fluidigm multiplexed assays. SNPs were selected based on minor allele frequency and presence on commercial genotyping platforms. SNP profiles are compared to SNP calls from available internal and external data (when available) to determine or confirm ancestry. In cases where data is unavailable or cell line ancestry is questionable, DNA or cell lines are re-purchased to perform profiling to confirm cell line ancestry. SNPs analyzed: rs11746396, rs16928965, rs2172614, rs10050093, rs10828176, rs16888998, rs16999576, rs1912640, rs2355988, rs3125842, rs10018359, rs10410468, rs10834627, rs11083145, rs11100847, rs11638893, rs12537, rs1956898, rs2069492, rs10740186, rs12486048, rs13032222, rs1635191, rs17174920, rs2590442, rs2714679, rs2928432, rs2999156, rs10461909, rs11180435, rs1784232, rs3783412, rs10885378, rs1726254, rs2391691,

rs3739422, rs10108245, rs1425916, rs1325922, rs1709795, rs1934395, rs2280916, rs2563263, rs10755578, rs1529192, rs2927899, rs2848745, rs10977980.

Mycoplasma Testing.

All stocks were tested for mycoplasma prior to and after cells were cryopreserved. Two methods were used to avoid false positive/negative results: Lonza Mycoalert kit and Stratagene Mycosensor. Cell growth rates and morphology were also monitored for any batch-to-batch changes.

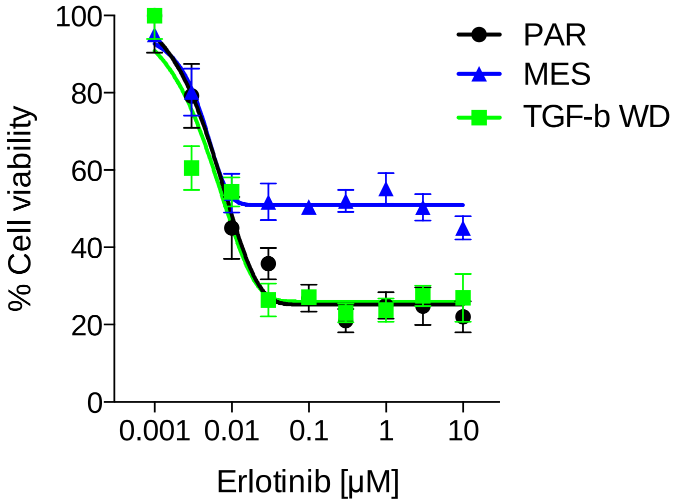

Supplementary Figure 1: Prolonged TGF-β withdrawal in mesenchymal cells are sensitive to erlotinib. Cell viability assay demonstrating the effect of erlotinib on upon withdrawal of TGF-β for 10 days (TGF-β WD) in HCC827 mesenchymal cells. Error bars represent mean ± SEM.

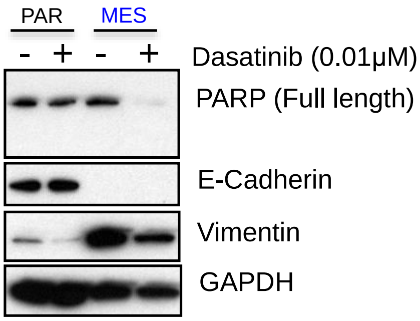

Supplementary Figure 2: TGF-β-induced EMT in A549 cells. Immunoblot demonstrating the expression of E-Cadherin, Vimentin and PARP in parental (PAR) and mesenchymal (MES) A549 cells following exposure to dasatinib (0.01μM) for 72 hours.

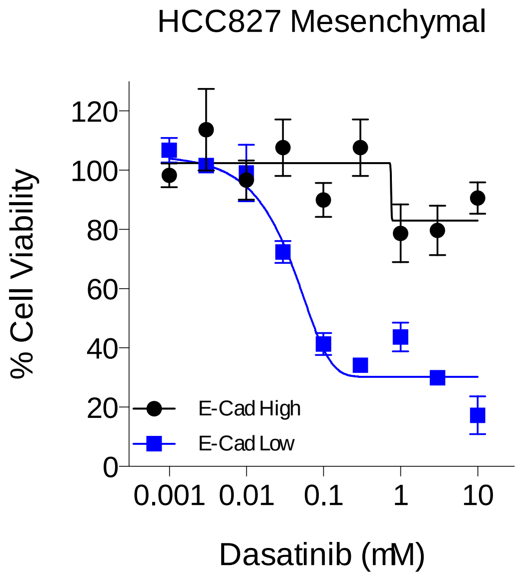

Supplementary Figure 3: Mesenchymal cells exhibit increased sensitivity to Dasatinib. Cell viability assay demonstrating the effect of dasatinib on E-Cadherin FACS-sorted high (E-Cad High) and low (E-Cad low) HCC827 mesenchymal cells. Error bars represent mean ± SEM.

Protein	Parental			Mesenchymal		
	DMSO	Dasatinib	Erlotinib	DMSO	Dasatinib	Erlotinib
CDK1	*20 (3)*	*45 (4)*	*9 (3)*	*71 (4)*	*79 (5)*	*52 (4)*
FAK1	*13 (7)*	*8 (4)*	*38 (17)*	*43 (22)*	*31 (15)*	*58 (27)*
CASL	*0 (0)*	*0 (0)*	*3 (1)*	*22 (9)*	*42 (14)*	*83 (19)*
AF1L2	*0 (0)*	*0 (0)*	*0 (0)*	*29 (11)*	*14 (9)*	*23 (13)*
MAP1B	*0 (0)*	*0 (0)*	*0 (0)*	*24 (12)*	*24 (14)*	*9 (6)*
FYN	*3 (2)*	*8 (4)*	*7 (4)*	*10 (6)*	*4 (3)*	*12 (7)*
MYH9	*2 (1)*	*9 (4)*	*8 (5)*	*5 (4)*	*7 (6)*	*9 (4)*
PTRF	*2 (1)*	*5 (3)*	*3 (2)*	*10 (4)*	*3 (2)*	*3 (2)*
ACK1	*0 (0)*	*1 (1)*	*2 (1)*	*5 (3)*	*8 (4)*	*7 (3)*
P85B	*0 (0)*	*0 (0)*	*2 (1)*	*5 (4)*	*6 (4)*	*6 (3)*
DOCK1	*0 (0)*	*0 (0)*	*0 (0)*	*3 (1)*	*0 (0)*	*13 (2)*
PTPRE	*0 (0)*	*0 (0)*	*0 (0)*	*4 (1)*	*0 (0)*	*5 (1)*
ABL1	*0 (0)*	*6 (2)*	*0 (0)*	*2 (2)*	*0 (0)*	*0 (0)*
CNN3	*0 (0)*	*0 (0)*	*0 (0)*	*3 (1)*	*0 (0)*	*0 (0)*
BCAR3	*0 (0)*	*0 (0)*	*0 (0)*	*0 (0)*	*0 (0)*	*2 (2)*
ACTN1	*0 (0)*	*0 (0)*	*0 (0)*	*0 (0)*	*0 (0)*	*1 (1)*

Supplementary Figure 4: Dasatinib suppresses Src/FAK signaling and associated protein phosphorylation in the mesenchymal cells. Table of peptide spectral matches for phosphotyrosine peptides following erlotinib (50nM) or dasatinib (30nM) treatment for 24h, demonstrating changes in phosphorylation in the HCC827 mesenchymal cells. Highlighted in red are those kinases associated with Src/FAK signaling.

Gene Name	Total (unique) peptides
FAK1	241 (47)
CASL	149 (28)
PEAK1	80 (15)
CDK1	68 (4)
AF1L2	60 (19)
MAP1B	57 (23)
P85B	49 (12)
PTRF	41 (7)
DOCK1	7 (1)
FYN	34 (8)
GIT2	32 (9)
MYH9	32 (6)
VCL	28 (4)
EPHB1	23 (5)
ACK1	31 (6)
IL31R	16 (4)
ACTN1	15 (5)
BCAR3	15 (6)
PTPRE	12 (1)
CNN3	9 (3)
ABL1	8 (4)

Supplementary Figure 5: Summary of tyrosine phosphorylated peptides identified in the HCC827 mesenchymal cells. Table summarizing peptide spectral matches (total and unique) observed following phosphotyrosine enrichment from HCC827 mesenchymal cells. Highlighted in red are those kinases associated with Src/FAK signaling.

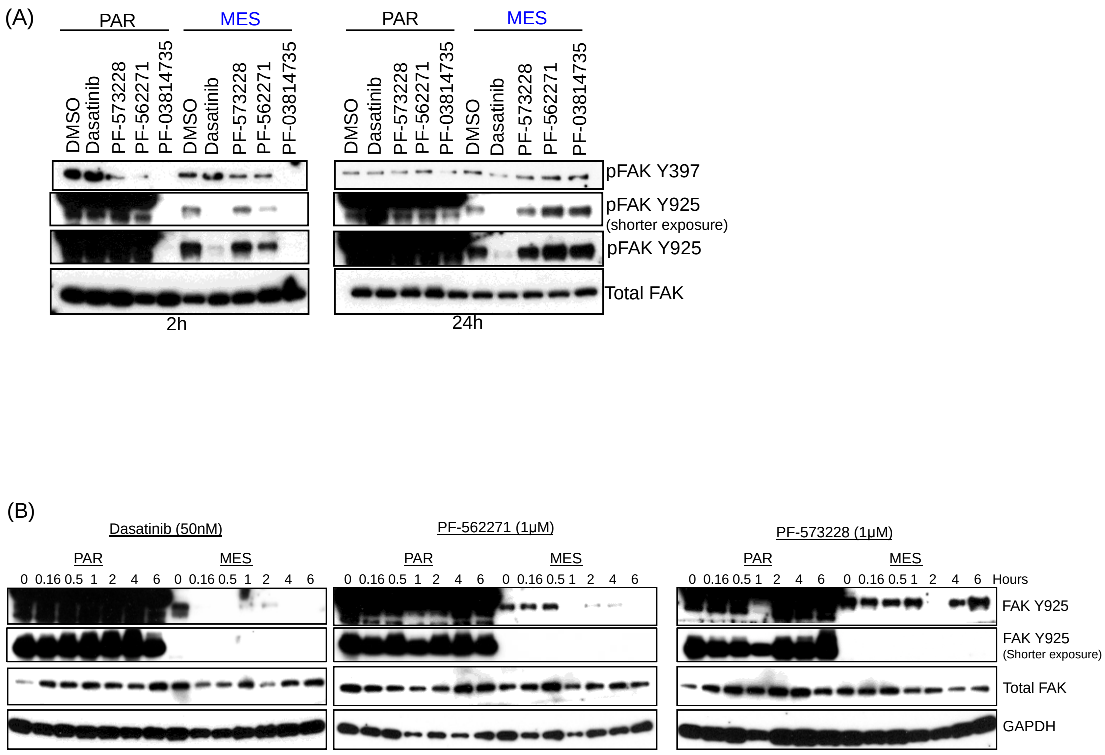

Supplementary Figure 6: Dasatinib suppresses pFAK more potently than reported FAK inhibitors. (A) Immunoblot demonstrating the expression of phospho-FAK in parental (PAR) and mesenchymal (MES) HCC827 cells following exposure to dasatinib (50nM), PF-562271 (1µM), PF-562271 (1µM) and PF-0381473 for 2 and 24 hours. (B) Immunoblot demonstrating the expression of phospho-FAK in parental (PAR) and mesenchymal (MES) HCC827 cells following exposure to dasatinib (50nM), PF-562271 (1µM), and PF-562271 (1µM) during a 6 hour time course.

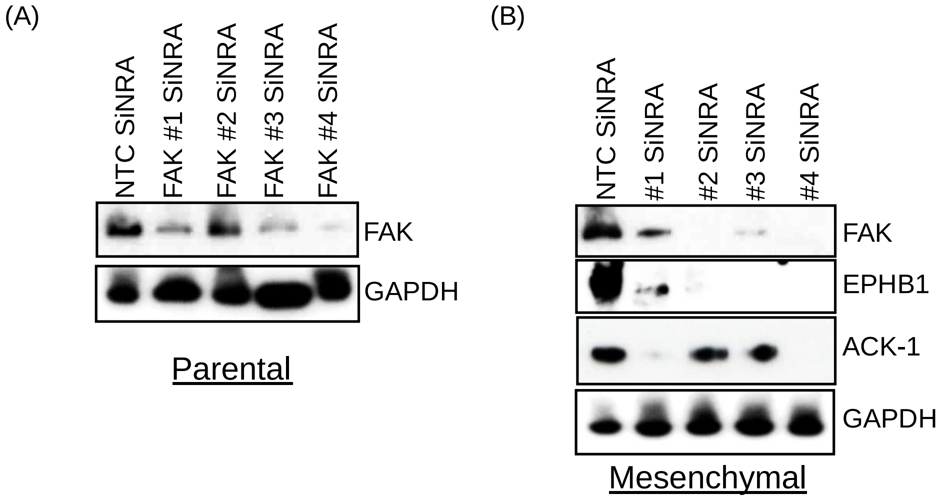

Supplementary Figure 7: SiRNA knockdown validation. (A) Immunoblot demonstrating FAK expression following siRNA for 72 hours in parental HCC827 cell line. (B) Immunoblot demonstrating FAK, EPHB1 and ACK-1 expression following siRNA for 72 hours in mesenchymal HCC827 cells.

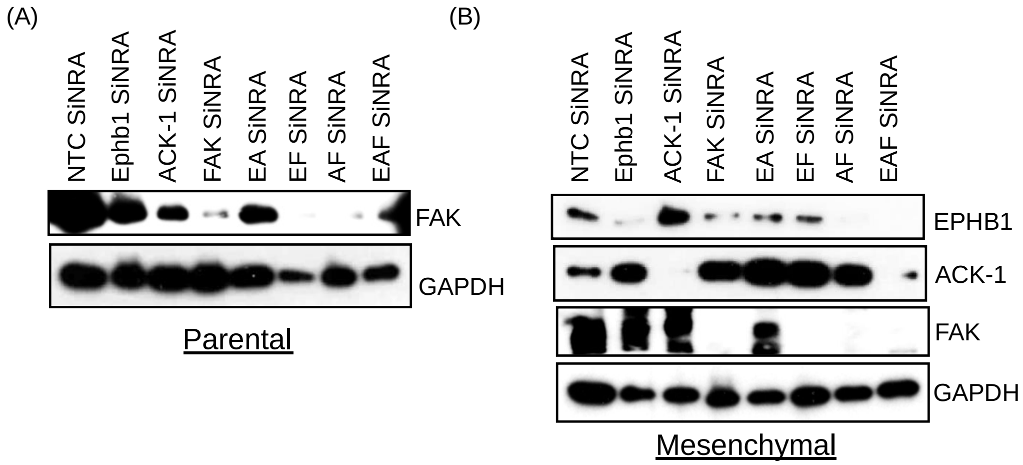

Supplementary Figure 8: SiRNA combination knockdown. SiRNA single or combined (EA; Ephb1&ACK-1, EF; Ephb1&FAK, AF; ACK-1&FAK, EAF; Ephb1,ACK-1& FAK) knockdown. SiRNA oligo#4 was used in all cases. (A) Immunoblot demonstrating FAK expression following siRNA for 72 hours in parental HCC827 cells. (B) Immunoblot demonstrating FAK, EPHB1 and ACK-1 expression following siRNA for 72 hours in mesenchymal HCC827 cells.

Table S1: Cell viability of HCC827 parental and mesenchymal cell lines showing IC_{50} values following 72h exposure to drug. Ratio reflects the IC_{50} of mesenchymal/parental cells.

Cell line: HCC827				
Drug	Target	PAR IC50 (μM)	MES IC50 (μM)	Ratio
Erlotinib	EGFR inhibitor	0.006	>10	1666.667
Gefitinib	EGFR inhibitor	0.003	>2	666.667
Docetaxel	Chemotherapeutic agent	0.032	>10	312.500
SB202190	p38 MAPK inhibitor	0.107	>20	186.916
Pictilisib	PI3K inhibitor	0.084	>10	118.747
DL11f	EGFR/HER3 inhibitor	0.132	>10	75.593
Trichostatin A	HDAC inhibitor	0.013	0.041	51.471
5-FU	Anti-metabolite	3.908	>200	51.177
PPP	IGF1R inhibitor	0.309	>10	32.362
MS-275	HDAC inhibitor	0.031	>1	32.258
GW 843682X	PLK inhibitor	0.795	>20	25.157
PD325901	MEK inhibitor	0.452	>10	22.124
BEZ235	PI3K inhibitor	0.560	>10	17.857
Salinomycin	Potassium ionophore	0.193	1.615	8.368
Mocetinostat	HDAC inhibitor	0.530	3.152	5.947
Doxorubicin	Chemotherapeutic agent	0.009	0.039	4.427
Lestaurtinib	JAK2 inhibitor	0.101	0.383	3.792
Cisplatin	Chemotherapeutic agent	3.382	>10	2.957
GF109203X	PKC inhibitor	3.394	>10	2.946
PF-562271	FAK/PYK2 inhibitor	3.455	>10	2.894
CGK733	ATM/ATR inhibitor	3.537	>10	2.827
Romidepsin	HDAC inhibitor	0.001	0.002	2.306
Sorafenib	VEGFR, PDGFR inhibitor	4.639	>10	2.156
SB220025	p38 MAPK inhibitor	2.743	5.673	2.068
Paclitaxel	Chemotherapeutic agent	0.015	0.031	2.067
Valproic acid	HDAC inhibitor	4.900	>10	2.041
PP2	SFK inhibitor	0.151	0.295	1.954
Flavopiridol	CDK2, CDK9 inhibitor	0.035	0.065	1.857
GW8510	CDK2 inhibitor	9.099	15.252	1.676
Crizotinib	MET/ALK inhibitor	3.810	5.198	1.364
PD173074	FGFR1/3 inhibitor	3.391	4.457	1.315
5-azacytidine	DNA Demethlyase	6.437	7.463	1.159
PKC412	EGFR T790M inhibitor	0.128	0.146	1.137
Imatinib	ABL1 inhibitor	17.603	>20	1.136
BV6	Dimer IAP antagonist	16.324	18.222	1.116
Vismodegib	Shh inhibitor	>10	>10	1.000
PF 573228	FAK inhibitor	>20	>20	1.000
Saracatinib	Abl/Src inhibitor	0.066	0.064	0.970
AEW541	IGF1R inhibitor	5.553	5.123	0.923
NSC625987	CDK4 inhibitor	22.840	>20	0.876
CPT-11/Irinotecan	Type 1 topoisomerase inhibitor	0.666	0.579	0.870
JQ1	Brd4 inhibitor	1.118	0.951	0.851
Lovastatin	inhibits HMG-CoA reductase	15.726	13.284	0.845
SAHA	HDAC inhibitor	1.300	1.000	0.769
5Z-7-Oxozeaenol	TAK1 inhibitor	3.391	2.543	0.750
H89	cAMP inhibitor	17.200	12.750	0.741
Vargatef	VEGF inhibitor	3.245	2.403	0.741
Phenformin	AMPK activator	0.265	0.185	0.698
Sunitinib	VEGF inhibitor	>10	5.650	0.565
BX912	PDK1 inhibitor	2.160	0.966	0.447
PF-03814735	Aurora kinase inhibitor	3.569	1.235	0.346
BAY 11-7821	NF-κB inhibitor	5.201	1.516	0.291
PHA-739358	Aurora kinase inhibitor	3.382	0.759	0.224
Dasatinib	Abl/Src inhibitor	0.062	0.009	0.145

Table S2: Cell viability of A549 parental and mesenchymal cell lines showing IC_{50} values of following 72h exposure to drug. Ratio reflects the IC_{50} of mesenchymal/parental cells.

Cell line: A549				
Drug	Target	PAR IC50 (µM)	MES IC50 (µM)	Ratio
GDC-0941	PI3K inhibitor	0.528	>20	37.864
Phenformin	AMPK activator	2.739	64.525	23.555
PD0325901	MEK inhibitor	0.013	0.105	8.211
Salinomycin	Potassium ionophore	0.087	0.478	5.494
5Z-7-Oxozeaenol	MEK inhibitor	0.642	3.371	5.255
5-FU	Chemotherapeutic agent	1.267	6.645	5.246
Carboplatin	Chemotherapeutic agent	26.674	>100	3.749
PD325901	MEK inhibitor	0.042	0.145	3.452
Pemetrexed	Chemotherapeutic agent	0.654	1.830	2.797
JQ1	Brd4 inhibitor	0.415	0.980	2.359
Gemcitabine	Chemotherapeutic agent	0.005	0.008	1.630
Erlotinib	EGFR inhibitor	0.820	1.252	1.527
Tunicamycin	Inhibits GPT	0.548	0.826	1.506
Gemcitabine	Chemotherapeutic agent	0.004	0.006	1.500
Flavopiridol	CDK2, CDK9 inhibitor	0.055	0.071	1.298
PKC412	EGFR T790M inhibitor	0.807	1.026	1.271
Sorefinib	VEGFR, PDGFR inhibitor	2.496	3.124	1.252
Imatinib	ABL1 inhibitor	5.793	7.082	1.223
Thapsigargin	Inhibitor of ER Ca++ ATPase	0.130	0.158	1.213
BEZ235	PI3K inhibitor	0.190	0.230	1.211
GW 843682X	PLK inhibitor	8.887	9.947	1.119
5-azacytidine	DNA Demethlyase	1.053	1.162	1.104
Nutlin-3a	p53/MDM2 inhibitor	3.364	3.652	1.086
Cytochalasin D	Chemotherapeutic agent	0.046	0.050	1.074
SAHA	HDAC inhibitor	1.634	1.747	1.069
PPP	IGF1R Inhibitor	0.244	0.250	1.025
PF-573228	FAK inhibitor	>20	19.720	0.986
PD173074	FGFR1/3 inhibitor	11.435	11.239	0.983
Bortezomib	Proteasome inhibitor	0.517	0.505	0.977
TAE684	ALK inhibitor	1.000	0.951	0.951
Doxorubicin	Chemotherapeutic agent	0.282	0.240	0.849
GW8510	CDK2 inhibitor	>20	15.704	0.785
PF-562271	FAK/PYK2 inhibitor	1.291	0.999	0.774
Lovastatin	inhibits HMG-CoA reductase	>20	14.604	0.730
Sunitinib	VEGF inhibitor	1.698	1.155	0.680
Mevastatin	inhibits HMG-CoA reductase	>20	13.431	0.672
SB431542	TGF-B inhibitor	3.187	2.095	0.657
Oligomycin	Inhibitor of OxPhos	0.105	0.062	0.585
Taxol	Chemotherapeutic agent	0.016	0.009	0.558
PF-03814735	Aurora kinase inhibitor	0.175	0.095	0.543
PF 573228	FAK inhibitor	4.525	2.422	0.535
Tivatinib	c-MET inhibitor	0.456	0.232	0.509
Crizotinib	c-MET/ALK inhibitor	0.856	0.315	0.368
PHA-739358	Aurora kinase inhibitor	0.703	0.257	0.366
AEW541	IGF1R Inhibitor	0.703	0.203	0.289
PP2	SFK inhibitor	4.354	0.890	0.204
BX912	PDK1 inhibitor	6.715	1.258	0.187
Dasatinib	Abl/Src inhibitor	0.614	0.022	0.036

Table S3: Listing of drugs used and their source.

Drug	Source
5-azacytidine	Sigma
5-FU	Sigma
5Z-7-Oxozeaenol	Tocris
AEW541	Selleck
BAY 11-7082	Selleck
BAY 11-7821	Tocris
BEZ235	SelleckBio
bortezomib	SelleckBio
BV6	GNE
BX912	Axon Medchem
Carboplatin	Sigma
CGK733	Tocris
Cisplatin	LC Laboratories
CPT-11/Irinotecan	LKT Laboratories
Crizotinib	LC Laboratories
Cytochalasin D	Tocris
Dasatinib	LC Laboratories
DL11f	GNE
Docetaxel	LC Laboratories
Doxorubicin	Sigma
Erlotinib	LC Laboratories
Flavopiridol	Sigma
GDC-0941	GNE
Gefitinib	SelleckBio
Gemcitabine	Toronto Research Chemicals
GF109203X	Tocris
GW 843682X	Axon Medchem
GW8510	Sigma
H89	Tocris
Imatinib	SelleckBio
JQ1	Selleck
Lestaurtinib	LC Laboratories
Lovastatin	Tocris
Mevastatin	Tocris
Mocetinostat	LC Laboratories
MS-275	Sigma
NSC625987	EMD
Nutlin-3a	Tocris
Oligomycin	Sigma
Paclitaxel	LC Laboratories
PD173074	Tocris
PD325901	Selleck
Pemetrexed	LC Laboratories
PF-03814735	Selleck
PF-562271	Selleck
PF573228	Tocris
PHA-739358	Selleck
Phenformin	Sigma
Pictilisib	LC Laboratories
PKC412	LC Laboratories
PP2	Tocris
PPP	Calbiochem
Romidepsin	Tocris
SAHA	Tocris
Salinomycin	Selleck
Saracatinib	LC Laboratories
SB202190	Calbiochem
SB220025	Calbiochem
SB431542	Selleck
Sorafenib	LC Laboratories
Sunitinib	Tocris
TAE684	Selleck
Taxol	Tocris
Thapsigargin	Tocris
Tivatinib	Selleck
Trichostatin A	Selleck
Tunicamycin	Tocris
Valproic acid	Calbiochem
Vargatef	LC Laboratories
Vismodegib	LC Laboratories
